# Comprehensive Comparison of Three Different Medicinal Parts of *Eupatorium lindleyanum* DC. Using the RRLC-Q-TOF-MS-Based Metabolic Profile and In Vitro Anti-Inflammatory Activity

**DOI:** 10.3390/molecules29153551

**Published:** 2024-07-28

**Authors:** Jiaojiao Lu, Chengbo Zheng, Simin Xue, Ye Gao, Guijin Chen, Chenxiao Shan, Ning Ding, Guoping Peng, Cunyu Li, Yunfeng Zheng

**Affiliations:** 1 Department of Pharmacy, Nanjing University of Chinese Medicine, Nanjing 210046, China; m18801587657@163.com (J.L.); chambo1015@163.com (C.Z.); xuesimin0912@163.com (S.X.); gaoye202359415@126.com (Y.G.); cgj2023523617@163.com (G.C.); chenxiao_shan@njucm.edu.cn (C.S.); dingning@njucm.edu.cn (N.D.); guopingpeng@126.com (G.P.); 2 National Key Laboratory on Technologies for Chinese Medicine Pharmaceutical Process Control and Intelligent Manufacture, Nanjing 211100, China; 3 Jiangsu Province Engineering Research Center of Classical Prescription, Nanjing University of Chinese Medicine, Nanjing 210023, China; 4 Jiangsu Collaborative Innovation Center of Chinese Medicinal Resources Industrialization, Nanjing University of Chinese Medicine, Nanjing 210023, China

**Keywords:** non-targeted plant metabolomics, structure identification, quantitative analysis, anti-inflammatory activity

## Abstract

*Eupatorium lindleyanum* DC. (EL) is a traditional Chinese herb known for its phlegm-reducing, cough-relieving and asthma-calming properties. It is widely used for treating cough and bronchitis. However, preliminary experiments have revealed wide variations in the composition of its different medicinal parts (flowers, leaves and stems), and the composition and efficacy of its different medicinal parts remain largely underexplored at present. In this study, non-targeted rapid resolution liquid chromatography coupled with a quadruple time-of-flight mass spectrometry (RRLC-Q-TOF-MS)-based metabolomics approach was developed to investigate the differences in the chemical composition of different medicinal parts of EL. We identified or tentatively identified 9 alkaloids, 11 flavonoids, 14 sesquiterpene lactones, 3 diterpenoids and 24 phenolic acids. In addition, heatmap visualization, quantitative analysis by high-performance liquid chromatography (HPLC-PDA) and ultra-high-performance liquid chromatography–triple quadrupole tandem mass spectrometry (UPLC-MS/MS) showed particularly high levels of sesquiterpene lactones, flavonoids and phenolic acids in the flowers, such as eupalinolide A and B and chlorogenic acid, among others. The leaves also contained some flavonoid sesquiterpene lactones and phenolic acids, while the stems were almost absent. The findings of in vitro activity studies indicated that the flowers exhibited a notable inhibitory effect on the release of the inflammatory factors TNF-α and IL-6, surpassing the anti-inflammatory efficacy observed in the leaves. Conversely, the stems demonstrated negligible anti-inflammatory activity. The variations in anti-inflammatory activity among the flowers, leaves and stems of EL can primarily be attributed to the presence of flavonoids, phenolic acids and sesquiterpene lactones in both the flowers and leaves. Additionally, the flowers contain a higher concentration of these active components compared to the leaves. These compounds mediate their anti-inflammatory effects through distinct biochemical pathways. The results of this study are anticipated to provide a scientific basis for the rational and effective utilization of EL resources.

## 1. Introduction

*Eupatorium lindleyanum* DC. (EL) boasts a long-standing tradition of use in Chinese medicine. Its aerial part, known as ‘Ye-ma-zhui’, is characterized by a bitter taste and being neutral in nature. It is associated with the lung meridian and is reputed for its effectiveness in resolving phlegm and relieving cough and asthma [1]. EL predominantly grows in Xuyi County, Jiangsu Province, and is a key component in the construction of standardized plant-based traditional Chinese medicinal materials in Jiangsu Province. It also plays a pivotal role in the pillar industry project for producing traditional Chinese medicinal materials in northern Jiangsu [2]. 

Recent pharmacological studies have revealed that EL has significant therapeutic effects for treating acute lung injury and upper respiratory tract infections. It can effectively reduce the production of pro-inflammatory mediators, lower the level of complement, inhibit the generation of oxygen radicals and decrease vascular permeability, thereby offering protection against acute lung injury [3]. The literature and preliminary research also indicate a spectrum of pharmacological effects of EL, encompassing lipid-lowering, anti-inflammatory, anti-viral and anti-cancer activities [4]. In clinical practice, EL is developed into tablets, syrup and compound capsules. Notably, EL syrup has been deemed safe and effective for treating chronic bronchitis in children [5], and compound capsules have shown significant efficacy in inhibiting the influenza virus and treating virus-induced pneumonia in mice [6]. Upper respiratory tract infection, a common, typically benign and self-limiting disease, has seen increasing patient numbers in recent years, with EL-related prescriptions demonstrating commendable clinical outcomes.

In the 2020 edition of the Chinese Pharmacopoeia, the medicinal part of EL is the above-ground part (i.e., the plant excluding the root). At present, these parts are commonly utilized in the production of various preparations, including EL syrup, EL tablets and compound EL capsules, etc. Cultivators generally prefer harvesting between August and October, although there is some debate about whether the peak flowering period is the most suitable time for harvesting. Hu et al. reported that the total flavonoid content and biomass per unit area of EL peaked during the initial blooming phase at the top of the flower head, suggesting that harvesting during this period could maximize the content of effective components and economic returns [7]. Conversely, Wang et al. found that the mineral content was generally higher in the stems and leaves than in the flowers, particularly in the tender stems and leaves harvested in April, proposing this as the optimal harvest time for medicinal efficacy [8]. Moreover, the literature and previous studies have identified the primary active ingredients in EL for treating upper respiratory tract infections and protecting against acute lung injury as sesquiterpene compounds (e.g., eupalinolide A and B) [3,9,10,11], whereas flavonoids (e.g., hyperoside) have been recognized as key for lowering blood pressure and lipids [12,13]. Preliminary experiments have, however, revealed substantial differences in the types and quantities of active components in the flowers, leaves and stems. Notably, the flowers exhibited a significantly higher diversity and concentration of active constituents compared to the leaves, while the stems contained the lowest amount of these compounds. Consequently, further investigations are warranted to optimize the harvesting period, identify the most efficacious medicinal parts and rationalize resource utilization with greater precision.

Building on preliminary research, a non-targeted RRLC-Q-TOF-MS-based metabolomics approach was developed to investigate the differences in the chemical composition of different medicinal parts of EL. Simultaneously, HPLC-PDA and UPLC-MS/MS methodologies were employed to quantitatively evaluate and confirm variances in the indicator components hyperoside, eupalinolide A, eupalinolide B, chlorogenic acid and echinatine. Furthermore, an in vitro model of chronic bronchitis was developed to examine the differential anti-inflammatory properties of the flowers, leaves and stems, thereby elucidating the correlation between their constituents and biological activities. This study seeks to establish a scientific basis for the rational and effective use of EL resources. It aims to offer insights and methodologies for conserving production resources during the medicinal material production process, thereby enhancing its clinical application.

## 2. Results

### 2.1. Identification of the Chemical Constituents of Different Medicinal Parts

**Figure 1:**
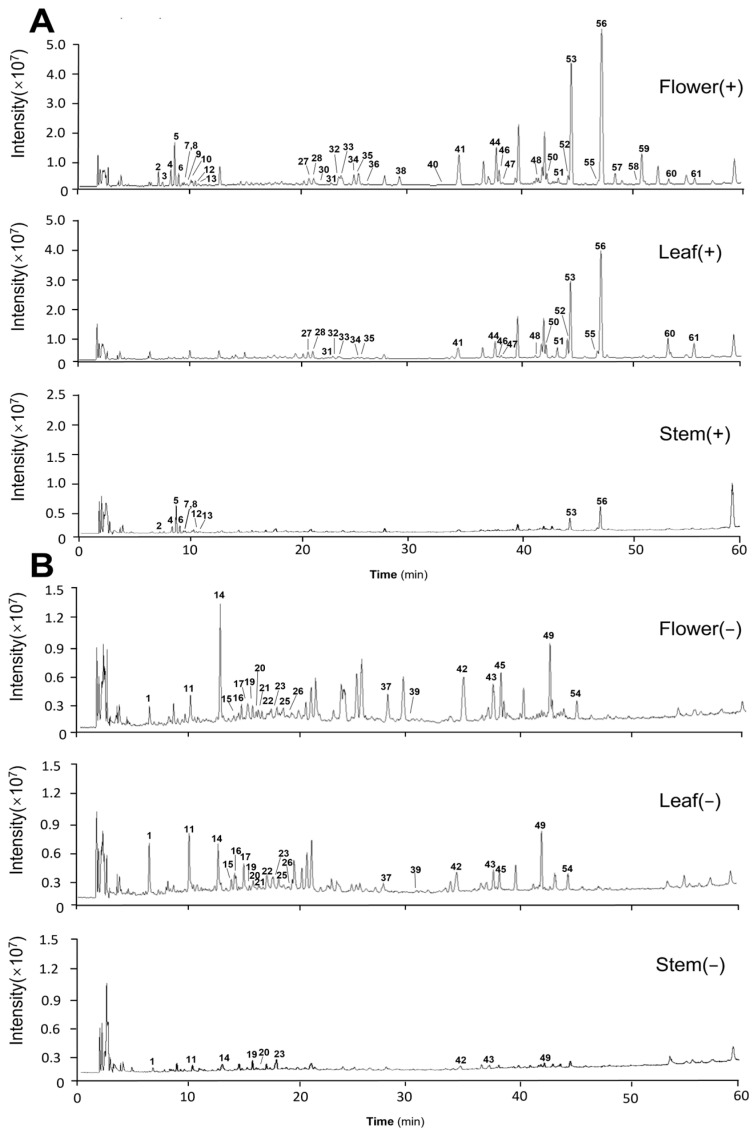
Full-scan total ion current (TIC) maps of positive and negative ion patterns of *Eupatorium lindleyanum* flower, leaf and stem samples. (**A**) Originated from the positive ion mode; (**B**) originated from the negative ion mode.

For a more comprehensive analysis of chemical constituents in the flowers, leaves and stems of EL, the positive and negative ion mode tests were implemented for RRLC-Q-TOF-MS analysis. The full-scan TICs for positive and negative ion modes of EL stem, leaf and flower samples, acquired under specified chromatographic and mass spectrometric conditions, are depicted in Figure 1A,B. The data were analyzed to identify targeted compounds with an error of less than 10 × 10^−6^, possessing an accurate isotopic distribution and containing secondary fragments. Using the online database of the software and the cleavage patterns of secondary fragments, we identified or preliminarily identified a total of 61 metabolites, including 9 alkaloids, 11 flavonoids, 14 sesquiterpenes, 3 diterpenes and 24 phenolic acids. Table 1 lists the detailed information of the identified compounds.

#### 2.1.1. Alkaloid Identification 

**Figure 2:**
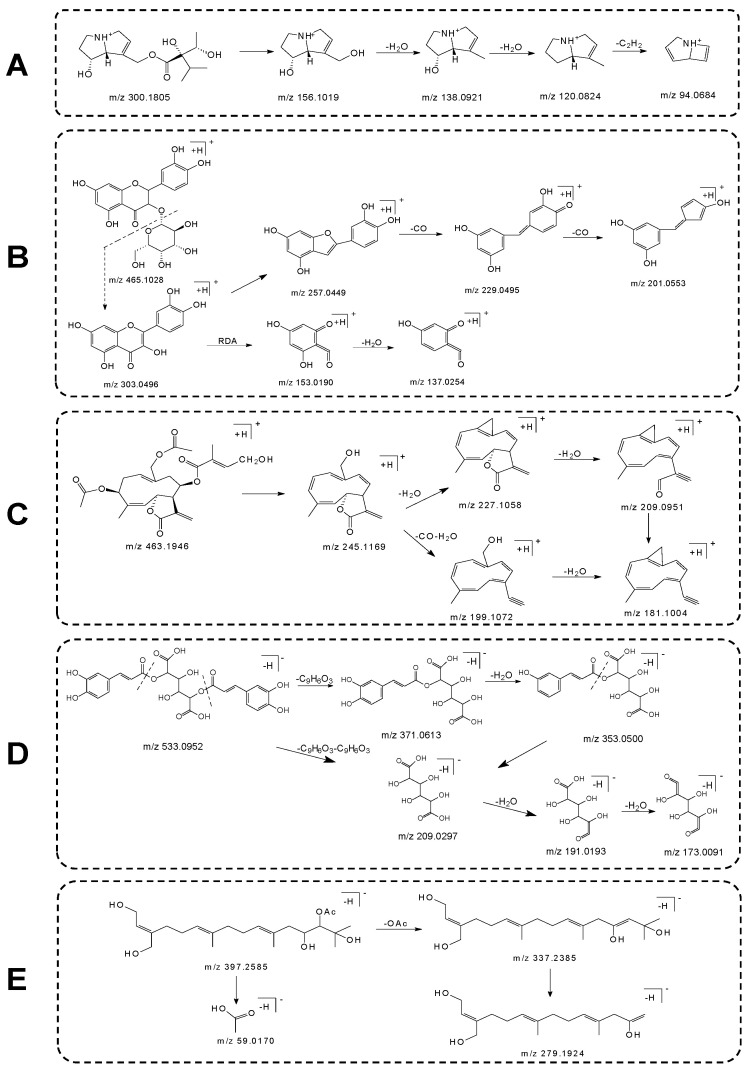
Cleavage pathways for 5 compounds. (**A**) The cleavage pathway of echinatine. (**B**) The cleavage pathway of hyperoside. (**C**) The cleavage pathway of eupalinolide A. (**D**) The cleavage pathway of 3,5-*O*-caffeoylquinic acid. (**E**) The cleavage pathway of 3-(hydroxymethyl)-1,14,15-trihydroxy-7,11,15-trimethyl-2,6,10-hexadecatrien-13-acetate.

In the analysis utilizing the positive ion mode, nine alkaloidal components (the compounds **2**–**7**, **9**, **12** and **13**) were identified. The compound **2** exhibited a retention time of 7.78 min, with its mass spectral peak for the [M+H]^+^ ion detected at *m/z* 300.1802. The characteristic ion and its corresponding secondary mass spectral information are depicted in the Supplementary Material (Figure S1). Through comparison with the retention times and mass spectral data of known standards, this compound was identified as echinatine, with its molecular formula established as C_15_H_25_NO_5_. Figure 2A illustrates the potential cleavage mechanism for echinatine. Both rinderine (**3**) and Intermedine (**12**), under the positive ion mode, displayed characteristic fragments at *m/z* 94, *m/z* 120 and *m/z* 138. These fragments are consistent with those of the echinatine standard, elucidating the carbon skeleton structure of the necine base group in 1,2-unsaturated PAs. The fragmentation patterns of N-oxides are similar to their non-oxidized counterparts, yet additional fragments could be observed. The oxygen located at the nitrogen atom could easily be cleaved by eliminating a water molecule (18 Da), thereby, in addition to the *m/z* 120 and *m/z* 138 fragments, fragments at *m/z* 136 and *m/z* 118 were also observed [14]. The compounds **4**, **5** and **6** in their secondary mass spectra exhibited peaks at *m/z* 172.0956, *m/z* 154.0854, *m/z* 138.0910 and *m/z* 136.0755, with the inferred secondary fragmentation ions corresponding to [M+H-C_7_H_12_O_3_]^+^, [M+H-C_7_H_12_O_3_-H_2_O]^+^ and [M+H-C_7_H_12_O_3_-H_2_O-O]^+^, respectively. The sequential reduction from *m/z* 172.0956 to *m/z* 138.0910 by 34 Da (H_2_O and O) suggests these compounds are likely N-oxides. Taking into account the retention times, molecular masses and secondary mass spectral data, it is theorized that the nine alkaloid compounds identified in *Eupatorium lindleyanum* belong to the class of pyrrolizidine alkaloids. This finding corroborates reports documented in the literature [15].

#### 2.1.2. Flavonoid Identification 

Within the ambit of positive ion mode analysis, a total of eleven flavonoid compounds were identified (comprised of compounds **27**, **28**, **30**, **31**, **32**, **33**, **34**, **36**, **38**, **47** and **51**). The compound **27**, through comparison with the retention times and mass spectral data of known standards, was identified as hyperoside, with its molecular formula deduced as C_21_H_20_O_12_. The ions and secondary mass spectra pertinent to this compound are depicted in the Supplementary Material (Figure S2), where fragment ions such as *m/z* 303.0496, *m/z* 257.0499 and *m/z* 229.0495 are observed. Figure 2B depicts the potential cleavage pathway of hyperoside. The compounds **27**, **28**, **33** and **36** were distinguished as isoflavonoids, discerned by their typical aglycone ions displayed at *m/z* 303.1220. These aglycone ions, through the loss of CO and H_2_O molecules, yield characteristic ions at *m/z* 257.0499, *m/z* 229.0495 and *m/z* 201.0553, while the Retro-Diels–Alder (RDA) reaction of the flavonoid skeleton leads to the generation of an ion at *m/z* 153.0190. From the quasimolecular ions and aglycone ions, it can be inferred that the molecular mass lost by the compounds corresponds either to glucose (162 Da) or malonyl-glucoside (248 Da). Isoquercitrin (**28**) was successfully identified through considerations of its molecular mass, cleavage pattern and comparisons against literature data [16]. The compounds **30**–**32**, **34** and **38** presented characteristic aglycone ions at *m/z* 287.0545, and based on the quasimolecular ions and aglycone ions, it is deduced that the loss corresponded to glucose (162 Da), malonyl-glucoside (248 Da), or rutinoside (308 Da). The compound **36** had a retention time of 25.92 min, with its mass spectral peak obtained at *m/z* 551.1011 and its molecular formula determined as C_24_H_22_O_14_. In the MS^2^ spectrum, the aglycone ion formed after the loss of malonyl-glucoside (248 Da) at *m/z* 303.1223 ([M+H-C_9_H_12_O_8_]^+^), alongside other characteristic ions at *m/z* 257.0413, *m/z* 165.0179 and *m/z* 85.0321 which were observed to be consistent with the MS^2^ spectrum of the compound **28**. Consequently, the compound **36** was identified as Quercetin 3*-O-*malonylglucoside.

#### 2.1.3. Sesquiterpene Lactones Identification 

Through the analysis conducted in positive ion mode, a total of fourteen sesquiterpene lactone compounds were identified (the compounds **41**, **44**, **46**, **48**, **50**, **52**, **53**, **55-61**). The compounds **53** and **56** were confirmed as eupalinolide A and eupalinolide B, respectively, through comparison with the retention times and mass spectral data of standard references. Eupalinolide A exhibited a retention time of 44.66 min, with its mass spectral peak for the [M+H]^+^ ion observed at *m/z* 463.1944, while the [M+NH_4_]^+^ and [M+Na]^+^ mass spectral peaks were detected at *m/z* 480.2212 and *m/z* 485.1769, respectively. The MS^2^ spectrum of eupalinolide A, as illustrated in the Supplementary Material (Figure S3), reveals four principal product ions. The [M+H]^+^ ion of this molecule underwent consecutive losses of a CH_3_COOH molecule, an OHC-CH molecule and a C_5_H_8_O_3_ molecule at *m/z* 463.1944, producing a signal at *m/z* 245.1169. Further, the formation of ions at *m/z* 227.1070, *m/z* 209.0962 and *m/z* 181.1006 ensued through the loss of one CO molecule and two H_2_O molecules. Figure 2C depicts several key fragmentation pathways of eupalinolide A. The molecular ions [M+H]^+^ of the compounds **44** and **50** were both positioned at *m/z* 419.1693, with the [M+Na]^+^ ion located at *m/z* 441.1497. The product ion at *m/z* 267.2083 suggests that these two compounds possess a 4-oxo-tigloyloxy substituent at the C-8 position and an OH substituent at the C-14 position. Based on the relevant literature, the compound **44** was identified as 3β-acetoxy-8β-(4′-oxo-tigloyloxy)-14-hydroxy-heliangolide, and the compound **50** as 3β-acetoxy-8β-(4′-oxo-tigloyloxy)-14-hydroxy-costunolide [17]. In positive ion mode, the compound **48** exhibited a mass spectral peak at *m/z* 421.1846 for the [M+H]^+^ ion at a retention time of 41.68 min, with a theoretical value of 421.1857, and its molecular formula was established as C_22_H_28_O_8_. MS^2^ spectral data revealed signals at *m/z* 245.1173, *m/z* 227.1062 and *m/z* 181.1015 formed through consecutive losses of a CH_3_COOH molecule, a C_5_H_8_O_3_ molecule, an H_2_O molecule and a CO molecule. Combined with the secondary mass spectral data and reference literature [18], the compound **48** was deduced to be eupalinolide F.

#### 2.1.4. Phenolic Acid Identification

In the analysis conducted under negative ion mode, twenty-four phenolic acids were successfully identified (the compounds **1**, **8**, **10**, **11**, **14-26**, **29**, **35**, **37**, **39**, **40**, **42**, **43**). Among these, the compound **14**, at a retention time of 12.82 min, displayed its [M-H]^−^ ion mass spectral peak at *m/z* 353.0882. Through a comparative analysis with the retention times and mass spectral data of control substances, this compound was confirmed to be chlorogenic acid, with its molecular formula determined as C_16_H_18_O_9_. The fragmentation process of chlorogenic acid with the molecular ion [M-H]^−^ at *m/z* 353.0882 preliminarily triggered by the loss of the caffeoyl group, subsequently generating the MS^2^ fragment ions of quinic acid at *m/z* 191.0572, and further yielding caffeic acid fragment ions at *m/z* 179.0358 through additional loss from quinic acid [19]. Similarly, the molecular ion peak [M-H]^−^ of the compound **10** was also *m/z* 353.0882, its MS^2^ spectrum indicated fragment ion signals at *m/z* 191.0572, *m/z* 179.0358 and *m/z* 135.0453, consistent with those of chlorogenic acid, thus leading to the characterization of this compound as neochlorogenic acid. The compounds **17**, **19** and **20** were all detected with [M-H]^−^ molecular ion peaks at *m/z* 533.0924 and their MS^2^ spectral chemical product fragment ions exhibited similarities, suggesting these three compounds as isomers. The extracted ion and secondary fragment data of the compound **17** can be found in the Supplementary Material (Figure S4), therein generating a signal at *m/z* 209.0301 through the consecutive losses of two C_9_H_6_O_3_ molecules from *m/z* 533.0924, followed by the formation of a signal at *m/z* 191.0193 due to the loss of an H_2_O molecule. Figure 2D elucidates its potential cleavage pathways. Based on related literature, it is deduced that these compounds are 3,5*-O-*caffeoylquinic acid, 1,5*-O-*caffeoylquinic acid and their isomers [20].

#### 2.1.5. Diterpene Identification 

In the mode of negative ions, three diterpene components were identified (the compounds **45**, **49**, **54**). Both the compounds **49** and **54** exhibited mass spectral peaks at *m/z* 397.2595 for [M-H]^−^, at *m/z* 433.2361 for [M+Cl]^−^ and at *m/z* 443.2650 for [M+FA-H]^−^. The extracted ion and secondary fragmentations for the compound **54** are depicted in the Supplementary Material (Figure S5), where the secondary mass spectrometry revealed chemical product fragment ions at *m/z* 337.2385, *m/z* 279.1924 and *m/z* 59.0170. At the [M-H]^−^ peak of *m/z* 397.2595, the loss of a CH_3_COOH molecule, as well as CH_3_ and H_2_O molecules, resulted in the signals at *m/z* 337.2385 and *m/z* 279.1924. It is conjectured that the compounds **49** and **54** are 3-(hydroxymethyl)-1,13,15-trihydroxy-7,11,15-trimethyl-2,6,10-hexadecatrien-14-acetate and 3-(hydroxymethyl)-1,14,15-trihydroxy-7,11,15-trimethyl-2,6,10-hexadecatrien-13-acetate, respectively. The cleavage pattern of the compound **49** is shown in Figure 2E. According to pertinent literature, the compound **45** is postulated to be 3-(hydroxymethyl)-1,12,14,15-tetrahydroxy-7,11,15,15-tetramethyl-2,6,10-hexadecatriene [21].

### 2.2. Analysis of Differential Composition of Different Medicinal Parts

#### 2.2.1. PCA and OPLS-DA Analysis

**Figure 3:**
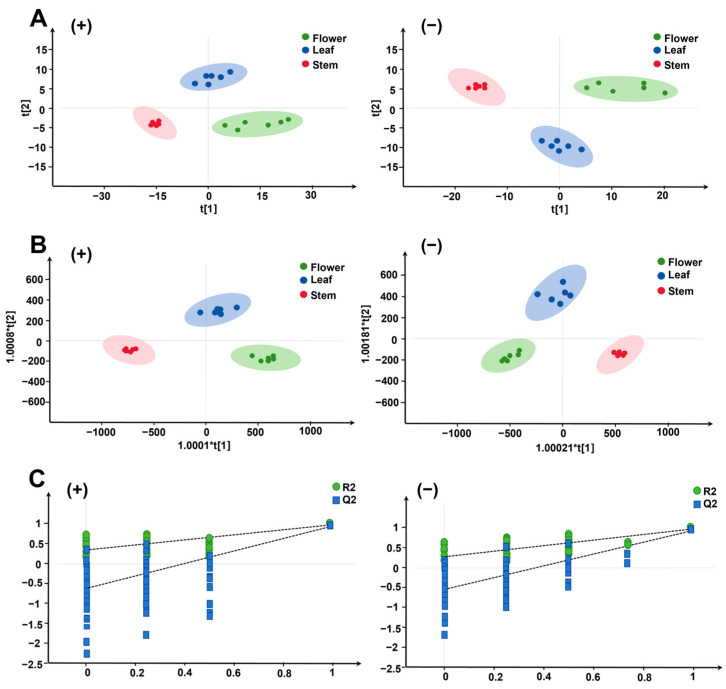
Principal component analysis (PCA) and orthogonal partial least squares analysis (OPLS-DA) of flowers, leaves and stems of 3 batches of Eupatorium lindleyanum. (**A**) PCA-X score plot. (**B**) OPLS-DA score plot. (**C**) Presentation of chance permutation at 200 times used for the discrimination among flowers, leaves and stems. (+) Originated from the positive ion mode; (−) originated from the negative ion mode.

Under optimized mass spectrometry conditions, 2271 positive ion pattern features and 2310 negative ion pattern features were extracted from all batches of flowers, leaves and stems. After normalization and filtering, 243 positive and 292 negative ions were isolated for PCA. The results of the unsupervised PCA analyses for six batches of flowers, leaves and stems with both positive and negative ion patterns are displayed in Figure 3A(+,−). Notable differences were observed between the three experimental groups: flowers were positioned to the left of the median, stems to the right and leaves in the center, closer to the flowers.

The LC-MS data were further analyzed using the orthogonal partial least squares discriminant analysis (OPLS-DA) model. The results of the OPLS-DA analysis of the positive and negative ion patterns for six batches of flowers, leaves and stems are shown in Figure 3B(+) and Figure 3B(−), respectively. To validate the OPLS-DA model, a replacement (prediction) test (*n* = 200) was performed. The results showed positive ions with R2Y (cum) = 0.982 and Q^2^ (cum) = 0.959 [Figure 3C(+)] and negative ions with R2Y (cum) = 0.973 and Q^2^ (cum) = 0.948 [Figure 3C(−)], indicating that the OPLS-DA model possesses robust identification and prediction capabilities. Chemometric analyses indicated a closer similarity in the chemical composition between flowers and leaves, as opposed to stems, which exhibited a greater dissimilarity from flowers and leaves, primarily attributable to their limited diversity and lower concentration of chemical constituents.

#### 2.2.2. Wayne Analysis of Differential Components

**Figure 4:**
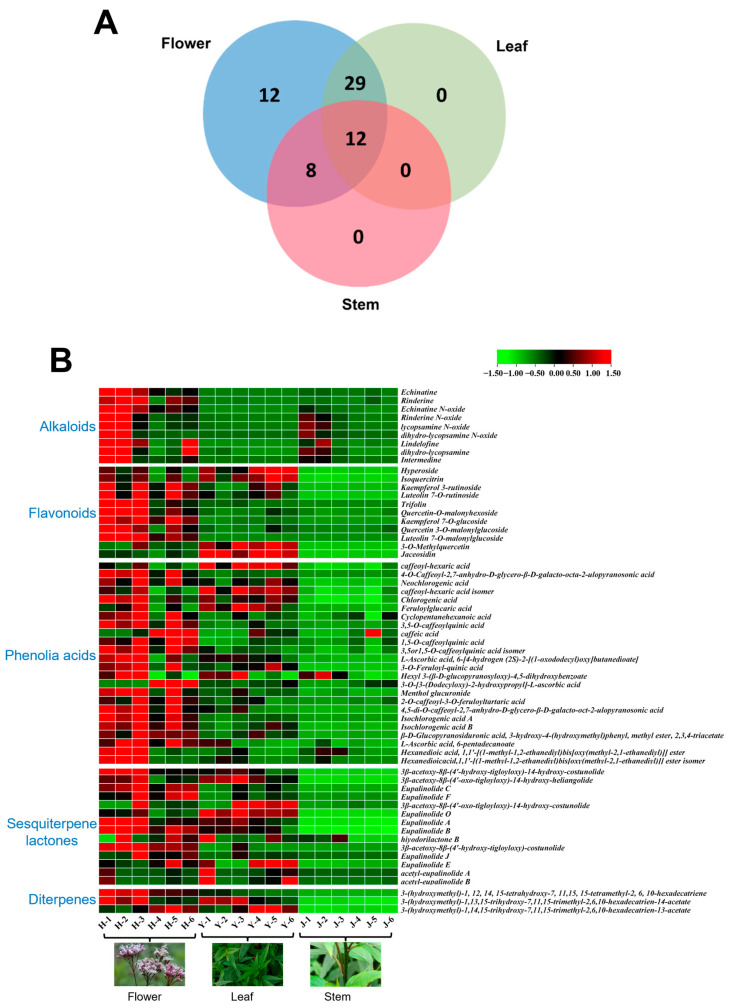
Compositional profiling of different medicinal parts of flowers, leaves and stems. (**A**) Wayne diagram analysis of the constituents of different medicinal parts. (**B**) Clustered heat map analysis of 61 components.

The compositional differences among the various medicinal parts were qualitatively depicted using Venn diagrams, as illustrated in Figure 4A. A comparative analysis of the constituents in flowers, leaves and stems, conducted through Venn diagram analysis, revealed 29, 8 and 0 overlapping constituents between flowers and leaves, flowers and stems and leaves and stems, respectively, across the three different medicinal parts. Among these, 12 constituents were common to all three parts. Furthermore, there were 12, 0 and 0 unique constituents in flowers, leaves and stems, respectively, and those endemic to the flowers include rinderine, lindelofine, quercetin-3*-O-*malonylglucoside, luteolin-7*-O-*malonylglucoside, neochlorogenic acid, caffeic acid, eupalinolide J and eupalinolide E and other alkaloids, phenolic acids, flavonoids and sesquiterpene lactones. The distribution of the components indicated that the constituents in flowers and leaves were more closely related, whereas the stems contained fewer components. Flavonoids, sesquiterpene lactones and phenolic acids were the predominant components in flowers and leaves, whereas alkaloids were more prevalent in flowers and stems.

#### 2.2.3. Heat Map Visualization of Component Spectra

To effectively illustrate the differences in component content among the different medicinal parts of EL, the peak areas of the 61 identified components were standardized, compared and presented in a heat map (Figure 4B). In this map, red indicates a higher content of components, whereas green signifies a lower content. As discernible from the figure, the alkaloids (the compounds **2**–**7**, **9**, **12** and **13**) were predominantly present in the flowers, with a minor presence in the stems and virtually absent in the leaves. The levels of phenolic acids (the compounds **1**, **8**, **10**, **11**, **14**–**26**, **29**, **35**, **37**, **39**, **40**, **42** and **43**), sesquiterpene lactones (the compounds **41**, **44**, **46**, **48**, **50**, **52**, **53** and **55**–**61**) and flavonoids (the compounds **30**, **31**, **32**, **33**, **34**, **36** and **38**) were significantly higher in the flowers compared to the stems and leaves, while stems were almost free of these components. It is noteworthy that the content of hyperoside, Isoquercitrin, 3*-O-*Methylquercetin, Jaceosidin (the compounds **27**, **28**, **47** and **51**) in the leaves were higher than that in the flowers. 

### 2.3. Quantitative Analysis of Four Representative Components by HPLC 

#### 2.3.1. Methodology Validation

Five differential components, hyperoside, eupalinolide A and B, chlorogenic acid and echinatine, were selected for quantitative analysis and differential validation. Among them, hyperoside, eupalinolide A, B and chlorogenic acid were higher in content and thus were further analyzed by HPLC-PDA (Figure S6A,B). The linear regression equations, linear ranges, correlation coefficients, limits of detection (LOD) and limits of quantification (LOQ) for these four compounds are detailed in Table 2. All calibration curves demonstrated strong linear relationships within the tested range. The RSD values for the precision of the four controls were below 1.06%, signifying the acceptable precision of the method. The RSD values for repeatability and stability were under 1.90%, indicating that the compounds remained essentially stable within 24 h. These results affirm that the method is suitable for quantifying these compounds. The total recovery rates for the spiked samples ranged from 98.77% to 107.90%, with an RSD of less than 2.33%, underscoring the accuracy and feasibility of the method.

#### 2.3.2. Determination of Content of Four Indicator Components

EL samples from flowers, leaves and stems were prepared into test solutions according to the method described in Section 2.2. Subsequently, the corresponding measurement methods outlined in Section 2.3 were employed to measure peak areas, and the content of each component was calculated using the standard curve method.

**Figure 5:**
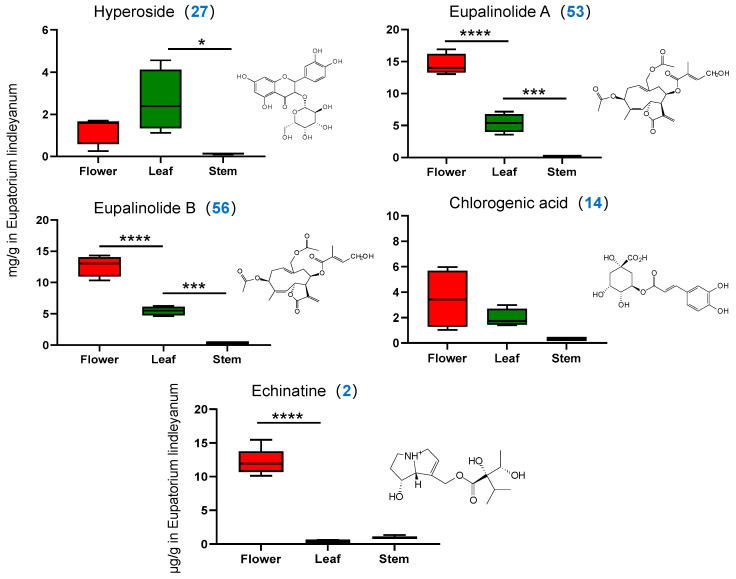
HPLC and UPLC-TQ-MS/MS with the MRM mode were used to analyze the content of five representative components in different medicinal parts. * *p* < 0.05, *** *p* < 0.001, **** *p* < 0.0001.

The Supplementary Material (Figure S6) presents HPLC and MRM chromatograms of five representative compounds obtained using HPLC-PDA and UPLC-MS/MS. The concentrations of the four compounds analyzed by HPLC-PDA in the three medicinal parts of EL, as depicted in Figure 5, exhibited significant variability. The average concentrations of hyperoside in flowers, leaves and stems were 1.283 ± 0.684, 2.617 ± 1.466 and 0.131 ± 0.037 mg/g, respectively, indicating that the concentration of hyperoside was higher in leaves than in flowers and stems. The concentrations of eupalinolide A and B were generally highest in the flowers, with average values of 14.494 ± 1.674, 5.390 ± 1.465 and 0.088 ± 0.040 mg/g for eupalinolide A and 12.681 ± 1.688, 5.469 ± 0.710 and 0.295 ± 0.082 mg/g for eupalinolide B across the three parts, respectively. Chlorogenic acid was predominantly found in flowers, followed by leaves, and was least present in stems. The three contents were 3.436 ± 2.311, 1.929 ± 0.710 and 0.279 ± 0.034 mg/g, respectively, which was consistent with the heat map visualization and provided differential validation of the metabolomics results.

### 2.4. Quantitative Analysis of One Alkaloidal Constituent by UPLC-MS/MS

#### 2.4.1. Methodology Validation

Further investigation of echinatine was conducted utilizing UPLC–MS/MS. The MRM parameters for echinatine were optimized using a 200 ng/mL single standard solution. The linear regression equations, linear range, correlation coefficient, LOD and LOQ for echinatine are presented in Table 2. Calibration curves demonstrated robust linear relationships within the test range. The RSD for precision regarding echinatine was 2.27%, signifying that the precision of the method is acceptable. The RSD values for repeatability and stability were 2.83% and 3.11%, respectively, indicating that the compound remained essentially stable over a 24 h period. The total spiked recovery was calculated at 102.18% with an RSD of 7.90%, underscoring the method’s accuracy and feasibility.

#### 2.4.2. Determination of Alkaloid Content

The peak area of each sample was determined in accordance with the method described in Section 2.5, and the content of each constituent was calculated using the standard curve method. As evident from Figure 5, the alkaloid content in the three medicinal parts varied significantly. The flowers had significantly more alkaloids at an average of 12.318 ± 1.817 ng/g compared to the leaves and stems, which had 0.390 ± 0.096 ng/g and 0.951 ± 0.204 ng/g, respectively. It is important to acknowledge that while the stems have a lower number of components, their alkaloid concentrations surpass those found in the leaves.

### 2.5. Study on the Anti-Inflammatory Activity of Different Medicinal Parts

#### 2.5.1. Modelling Chronic Bronchitis In Vitro

**Figure 6:**
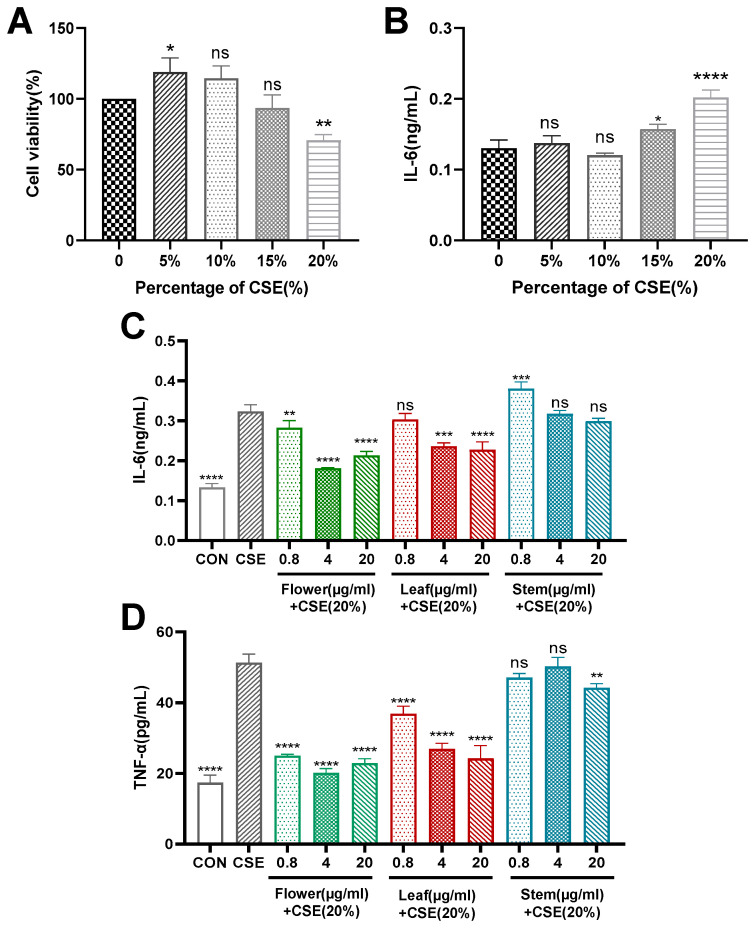
Establishment of a model of chronic bronchitis and changes in inflammatory factors in cell supernatants. (**A**) The effect of CSE on cellular activity. (**B**) The effect of CSE on cellular IL-6 expression. (**C**) The level of IL-6 in cell supernatants. (**D**) The level of TNF-α in cell supernatants. Results are shown as the mean ± SEM. (n ≥ 3, ns for no significance, * *p* < 0.05, ** *p* < 0.01, *** *p* < 0.001, **** *p* < 0.0001 compared to control).

The 16HBE cells were divided into a control group and groups exposed to different concentrations of CSE (5%, 10%, 15% and 20%). The cells were stimulated with CSE for 24 h. Compared to the control group, CSE initially increased cell activity and subsequently decreased it. Notably, the 20% concentration of CSE significantly reduced cell viability (Figure 6A). The expression level of the inflammatory factor IL-6 in the cell supernatant was measured using ELISA. The results indicated a significant increase in IL-6 under the stimulation of 20% CSE (Figure 6B), with a statistically significant difference.

#### 2.5.2. Flower, Stem and Leaf Extracts Reduce CSE-Induced IL-6 and TNF-α Expression in 16HBE Cells

Cells were pre-treated with different concentrations of flower, stem and leaf extracts for 2 h, followed by the addition of 20% CSE for 24 h. ELISA was used to measure the expression levels of IL-6 (Figure 6C) and TNF-α (Figure 6D). The findings showed that high-dose flower extracts significantly diminished the CSE-induced expression of IL-6 and TNF-α in 16HBE cells. The efficacy of the three extracts in reducing the expression levels of inflammatory factors was in the order of flower > leaf > stem. 

## 3. Discussion

In this study, a non-targeted metabolomics approach employing RRLC-Q-TOF-MS was used to efficiently analyze the chemical constituents of three medicinal parts of EL, namely the flower, leaf and stem. A total of 61 metabolites including sesquiterpene lactones, alkaloids, phenolic acids, flavonoids and diterpenes were identified by reference substances and their characteristic fragmentation spectra. Through multivariate analysis, heatmap visualization and quantitative analysis based on HPLC-PDA and UPLC-MS/MS, notable variations in metabolite type and content were observed among different medicinal parts. Specifically, flowers exhibited higher levels of sesquiterpene lactones, flavonoids and phenolic acids compared to leaves, and the contents of eupalinolide A and B, as well as chlorogenic acid, were found to be two to four times greater in flowers than in leaves. The concentration of some flavonoids in leaves was high, with hyperoside levels in leaves being two to three times greater than in flowers. Conversely, the presence of these components in stems was minimal. Alkaloids were predominantly present in flowers and stems. In order to further study the pharmacodynamic differences among different medicinal parts of EL, their anti-inflammatory activities were compared.

Cigarette smoke (CS) exposure is closely associated with bronchitis and inflammation. CS impairs airway epithelial function, mucociliary clearance and innate immune responses, leading to airway inflammation and fibrosis and promoting the release of various inflammatory mediators [22], including tumor necrosis factor α (TNF-α), macrophage inflammatory protein-2 (MIP-2) and myeloperoxidase (MPO) [23]. This study utilized a model of CSE-induced inflammation in human bronchial epithelial cells to compare the anti-inflammatory properties of three different medicinal parts. The findings of the study demonstrated that the flowers significantly inhibited the secretion of the inflammatory factors TNF-α and IL-6. The anti-inflammatory effect of the leaves was comparatively weaker, and the stems exhibited minimal anti-inflammatory activity. This outcome can be attributed to the varying compositions of the three medicinal parts. Previous research has suggested that sesquiterpene lactones may be effective in treating acute lung injury and upper respiratory tract infections by suppressing the release of inflammatory mediators. Zhong et al. demonstrated that sesquiterpenoids derived from EL containing a lactone ring structure, such as eupalinolide A, B, L and M, exhibited anti-inflammatory effects by suppressing the secretion of TNF-α and IL-6 from inflammatory cells [10]. Additionally, Huang et al. demonstrated that eupalinolides C and K exhibit potent anti-inflammatory properties, significantly inhibiting the inflammatory factor IL-6. However, the underlying mechanisms of these effects warrant further investigation [11]. The anti-inflammatory properties of polyphenols are attributed to their capacity to safeguard cells from oxidative harm. Dan et al. demonstrated that CSE can induce the nuclear translocation of nuclear factor erythroid 2-related factor 2 (Nrf2) and upregulate the expression of NAD(P)H: quinone oxidoreductase 1 (NQO1) and heme oxygenase 1 (HO-1). Concurrently, CSE activates mitogen-activated protein kinase (MAPK) signaling pathways, resulting in oxidative cellular damage and subsequent airway inflammation [24]. Conversely, isoquercetin exhibits a protective effect against apoptosis and oxidative damage by inhibiting the Nrf2/HO-1/NQO1 pathway [25]. In comparison to quercetin, isoquercetin has a higher ROS scavenging activity and protects cells from reactive oxygen species (ROS)-induced oxidative damage [26]. Likewise, chlorogenic acid demonstrates anti-inflammatory properties through the inhibition of the NF-κB and MAPK signaling pathways [27]. In conclusion, the notable anti-bronchitis activity exhibited by flowers and leaves can be attributed to their abundant presence of sesquiterpene lactones, flavonoids and phenolic acids. These compounds demonstrate promising anti-inflammatory effects by modulating various pathways, including the suppression of inflammatory mediator release, inhibition of ROS production and mitigation of oxidative stress. Flowers, in particular, exhibit enhanced anti-inflammatory properties compared to leaves, owing to their higher concentration of bioactive constituents.

Furthermore, our experiments revealed a higher concentration of pyrrolizidine alkaloids (PAs) in the flowers of EL, significantly exceeding the levels in stems and leaves. Despite the stems containing fewer components, their concentration of these alkaloids surpasses that in the leaves. According to the existing literature, PAs are predominantly hepatotoxic [14,28,29] and excessive consumption could increase the risk of hepatic veno-occlusive disease. In addition, various cellular and animal studies suggest that some PAs may cause damage to the brain and lungs [30]. With growing concerns over the safety of traditional Chinese medicine, assessing the safety of EL flowers used medicinally becomes critical. Consequently, we could consider PAs as a key indicator for quality control and establish stringent standards to limit their content in medicinal materials, thereby reducing potential toxic effects. Simultaneously, future research should focus on developing and optimizing production and processing techniques to selectively remove PAs, ensuring the sustainable use and safety of EL resources.

## 4. Experimental Section

### 4.1. Plant Material, Standards and Reagents

The EL samples were divided into six batches, which were sourced from the medicinal botanical garden of Nanjing University of Traditional Chinese Medicine (NJUCM) and the medicinal material planting area of Xuyi County, Jiangsu Province. Professor Yan Hui of NJUCM confirmed that these samples were the dried aerial parts of EL. The flowers, stems and leaves were separated, dried at 60 °C, weighed and ground to produce powdered samples of the different parts (stems, leaves, flowers) of EL. The batch numbers corresponding to the herbs are detailed in Table 3.

The study utilized five standard products. The reference standard for hyperoside was obtained from Nanjing Jinyibai Biotechnology Co., Ltd., Nanjing, China (JBZ-0554, purity 98.45%). The laboratory synthesized the reference standards for eupalinolide A and eupalinolide B, achieving a purity of >98%. The reference standard for chlorogenic acid was obtained from Shanghai Bide Pharmaceutical Technology Co., Ltd. (BD33230-25g, purity 99.58%), and the echinatine reference standard from Chengdu Pusi Biotechnology Co., Ltd., Chengdu, China (PS230621-10, purity ≥98%). Methanol and formic acid of HPLC grade and acetonitrile of both HPLC and MS grade were purchased from Merck (Darmstadt, Germany). Ultra-pure water was procured using the Milli-Q purification system (Millipore, Milford, MA, USA).

The human bronchial epithelial cell line (16HBE) was acquired from Wuxi Dudi Biotechnology Co., Ltd., Wuxi, China; RPMI-1640 medium (containing 1% penicillin/streptomycin) and phosphate-buffered saline were sourced from Nanjing Kaiji Biotechnology Development Co., Ltd., Nanjing, China, Fetal bovine serum (FBS) was obtained from Zhejiang Tianhang Biotechnology Co., Ltd., Huzhou, China. The MTT solution was purchased from Shanghai Yuanye Biotechnology Co., Ltd., Shanghai, China, and ELISA assay kits from Nanjing Jinyibai Biotechnology Co., Ltd., Nanjing, China.

### 4.2. Sample Preparation

For HPLC analysis, approximately 0.5 g of the powdered flowers, leaves and stems of EL was accurately weighed and transferred into a conical flask. To this, 25 mL of 50% methanol was accurately added. The mixture was weighed, sonicated for 30 min and weighed again to compensate for any weight loss. After thorough mixing, the sample was centrifuged at 10,000 rpm for 5 min. Next, the supernatant was collected for HPLC and RRLC-Q-TOF-MS analyses. The filtrates of the flowers, leaves and stems were diluted with a 50% methanol–water solution at ratios of 100:1, 10:1 and 20:1, respectively, and subsequently injected into the UPLC–MS/MS system for quantitative analysis.

For the anti-inflammatory activity assay, approximately 20 g of powdered EL flowers, leaves and stems of EL was used. Each sample was subjected to ultrasonic extraction using eight times its weight of 60% ethanol solution for 30 min. After the first extraction, the residue was filtered and then extracted again with six times its weight of 60% ethanol solution for an additional 30 min. The filtrates from both extractions were combined and concentrated under reduced pressure at 50 °C before being freeze-dried. This process yielded separate extracts of the flower, leaf and stem, weighing 1.94 g, 2.15 g and 0.84 g, respectively.

### 4.3. Preparation of Standard Solutions

Five separate standard solutions of five compounds were prepared in a 50% methanol–water solution. Appropriate volumes of these standard solutions were transferred to 10 mL volumetric flasks and diluted with a 50% methanol solution to form mixed standard solutions. The concentrations of the mixed standard solution for HPLC analysis were as follows: hyperoside at 0.22 mg/mL, eupalinolide A at 0.53 mg/mL, eupalinolide B at 0.51 mg/mL and chlorogenic acid at 0.48 mg/mL. Working standard solutions for the calibration curve were prepared using the gradient dilution method. For LC-Q-TOF-MS analysis, the concentrations of the reference standards were as follows: hyperoside at 23 µg/mL, eupalinolide A at 29 µg/mL, eupalinolide B at 27.5 µg/mL, chlorogenic acid at 31 µg/mL and echinatine at 30 µg/mL. A working standard solution of echinatine at 170 ng/mL was prepared, and further working standard solutions for the calibration curve were prepared using the gradient dilution method for quantitative analysis in UPLC–MS/MS.

### 4.4. HPLC Chromatographic Conditions

Chromatography was conducted using a Hedera C18 column (250 mm × 4.6 mm, 5 µm). The mobile phases comprised solvent A (acetonitrile) and solvent B (water containing 0.1% formic acid). The gradient program was set as follows: 0–15 min, 5–20% A; 15–30 min, 20–25% A; 30–40 min, 25–40% A; 40–60 min, 40–60% A. The flow rate was maintained at 1 mL/min. Detection wavelengths were set at 361 nm and 210 nm. The column temperature was maintained at 30 °C, and the injection volume was 10 µL.

### 4.5. RRLC-Q-TOF-MS Spectrometric Conditions

The chromatographic conditions adhered to those outlined in Section 2.4, with a modification in the injection volume, which was set at 5 µL. The ionization conditions were established as follows: the system operated in positive–negative ionization mode with spray voltages of +5500 V and −4500 V. The nebulizer pressure was set at 0.35 kPa, the curtain pressure at 0.15 kPa and auxiliary gas pressure at 0.30 kPa. The ionization temperature was maintained at 550 °C. The cluster cracking voltage was 65 V, and the collision energy was 45 V. The detection mode employed was IDA.

### 4.6. UPLC-TQ-MS/MS Spectrometric Conditions

Chromatographic analysis was conducted using an LC-20CE XR ultra-fast liquid chromatography system (Shimadzu, Kyoto, Japan) equipped with a SIL-20A XR autosampler and a CTO-20AC column oven. The chromatographic column employed was a Waters ACQUITY UHPLC HSS T3 column (2.1 × 150 mm, 1.8 μm), maintained at a temperature of 30 °C. The mobile phase for UPLC–MS/MS comprised solvent A (acetonitrile) and solvent B (water with 0.1% formic acid), eluted with 10% acetonitrile isocratic elution, a flow rate set to 0.3 mL/min and an injection volume of 1 μL. Quantitative analysis of representative compounds was executed using a triple quadrupole linear ion trap mass spectrometer (QTRAP 5500 system, AB SCIEX). Compound-specific mass parameters were designed and optimized employing Applied Biosystems/MDS SCIEX Analyst software (version 1.6.3).

### 4.7. Study on the Anti-Inflammatory Activity of Different Medicinal Parts

#### 4.7.1. Cell Cultures 

Human bronchial epithelial cells (16HBE) were cultured in RPMI-1640 medium supplemented with 10% FBS and 1% penicillin/streptomycin. The cells were incubated under ambient conditions of 37 °C and 5% CO_2_.

#### 4.7.2. Preparation of Cigarette Smoke Extract (CSE) 

CSE was prepared following the method previously described [31]. In brief, two cigarettes (NJ) were smoked continuously for 3 to 5 min, and the smoke was collected using a peristaltic pump device. Subsequently, the smoke was bubbled slowly into 10 mL of RPMI-1640 medium. The pH of the CSE solution was then adjusted to 7.2, and a filter with a pore size of 0.22 μm was employed to remove bacteria and large particles. This solution was considered 100% CSE and was subsequently diluted to various concentrations for the ensuing experiment.

#### 4.7.3. In Vitro Chronic Bronchitis Modelling 

The 16HBE cells were seeded at a density of 10,000 cells per well in 96-well plates. Following a 12 h incubation period, the spent medium was replaced with fresh medium containing varying concentrations of CSE, specifically 5%, 10%, 15% and 20%. After an additional 24 h of incubation under normal conditions, cell viability was assessed using an MTT assay. Furthermore, the expression level of IL-6 in the cell supernatant was quantified using an ELISA.

#### 4.7.4. ELISA for Inflammatory Factor Expression 

The 16HBE cells were seeded at a density of 10,000 cells per well in 96-well plates. Following a 12 h incubation period, the cells were pretreated with different concentrations of flower, stem and leaf extracts for 2 h. Subsequently, 20% CSE was added, culminating in a total treatment duration of 24 h. Cell supernatants were then collected and centrifuged at 4 °C at 3000 rcf for 15 min. The supernatants were carefully collected, and the expression levels of IL-6 and TNF-α were quantified according to the detailed procedures provided in the ELISA kit instructions.

### 4.8. Data Processing and Statistical Analyses

Mass spectrometry spectra were analyzed using PeakView 1.2 (AB Sciex, Foster City, CA, USA) to obtain the total ion chromatograms (TIC) of samples from different medicinal parts of EL, in both positive and negative ion modes. This analysis facilitated the determination of the retention times and secondary mass spectrometry data for each constituent. The various chemical constituents were identified by referencing relevant literature.

LC-Q-TOF-MS data from different medicinal parts of EL were read and preliminarily analyzed in both positive and negative ion modes using MarkerView 1.2.1 (AB Sciex, Foster City, CA, USA). The intensity of each ion was normalized and filtered relative to the total ion counts to generate a data matrix. This matrix included *m/z* values, retention times and normalized peak areas. For calibration across different samples, the mass accuracy was set to ±10 ppm, the retention time range was established from 5 to 60 min, the retention time difference was adjusted to ±0.20 min and an intensity threshold of 5000 counts was applied. In addition, isotopic peaks were excluded from the analysis.

The processed data were imported into SIMCA 13.0 (Umetrics, Umea, Sweden) for pattern recognition analysis [32]. The dataset underwent preprocessing, which involved centering on the mean and scaling using Pareto (Par). Subsequently, two commonly used data dimensionality reduction methods in pattern recognition were applied: principal component analysis (PCA) and orthogonal partial least squares discriminant analysis (OPLS-DA). The reliability of the assessment models was determined using replacement test methods. The R2X and R2Y values described the predictive performance of the OPLS-DA models.

Data from the anti-inflammatory activity experiments were statistically analyzed using GraphPad Prism 9.0 software. This analysis included data from at least three independent replications of the experiments. The results were expressed as the mean ± SEM, and one-way ANOVA was utilized to compare groups. Differences were considered statistically significant when *p* < 0.05.

## 5. Conclusions

In conclusion, the study determined that among the three medicinal components of EL, the flower exhibits richer compounds and demonstrates superior anti-inflammatory activity. These findings offer a scientific foundation for optimizing the harvesting period and enhancing the rational and effective utilization of EL resources. Simultaneously, they offer insights and methodologies for the production process of medicinal herbs, aimed at conserving production resources and enhancing their efficacy in clinical applications.

## Figures and Tables

**Table 1:** Data of compounds identified from Eupatorium lindleyanum DC. according to RRLC-Q-TOF-MS.

NO.	RT (min)	Protonated Molecular Ion	Measured (*m*/*z*)	Characteristic Fragment Ions	Error (ppm)	Molecular Formula	Identification	Stem	Leaf	Flower
1	6.52	[M-H]^−^	371.0624	209.0318, 191.0195, 179.0352, 135.0452, 85.0307	1.1	C_15_H_16_O_11_	Caffeoyl-hexaric acid	+	+	+
2	7.31	[M+H]^+^	300.1804	156.1017, 138.0917, 120.0814, 94.0617	−0.5	C_15_H_25_NO_5_	Echinatine *	+	−	+
3	7.67	[M+H]^+^	300.1804	156.1018, 138.0918, 120.0813, 94.0672	−0.5	C_15_H_25_NO_5_	Rinderine	−	−	+
4	8.43	[M+H]^+^	316.1754	172.0964, 155.0943, 154.0864, 138.0918, 136.0762	−0.2	C_15_H_25_NO_6_	Echinatine N-oxide	+	−	+
5	8.77	[M+H]^+^	316.1755	172.0956, 155.0932, 154.0854, 138.0910, 136.0755	0.1	C_15_H_25_NO_6_	Rinderine N-oxide	+	−	+
6	9.13	[M+H]^+^	316.1755	172.0973, 155.0948, 138.0924, 136.0770	0.1	C_15_H_25_NO_6_	Lycopsamine N-oxide	+	−	+
7	9.48	[M+H]^+^	318.1909	174.1126,156.1020,113.0848,82.0686	−0.7	C_15_H_27_NO_6_	Dihydro-lycopsamine N-oxide	+	−	+
8	9.69	[M-H]^−^	397.0776	235.0450, 179.0342, 161.0244, 135.0452, 133.0295	−0.1	C_17_H_18_O_11_	4-o-Caffeoyl-2,7-anhydro-d-glycero-β-d-galacto-octa-2-ulopyranosonic acid	+	−	+
9	9.87	[M+H]^+^	286.2013	142.1232, 124.1131, 96.0832	−1.0	C_15_H_27_NO_4_	Lindelofine	−	−	+
10	10.04	[M-H]^−^	353.0882	191.0572, 179.0358, 135.0453	1.1	C_16_H_18_O_9_	Neochlorogenic acid	−	−	+
11	10.18	[M-H]^−^	371.0625	209.0299, 191.0204, 85.0307	1.4	C_15_H_16_O_11_	Caffeoyl-hexaric acid isomer	+	+	+
12	10.38	[M+H]^+^	302.1959	158.1173, 141.1149, 140.1074, 124.1130, 110.0978	−1.0	C_15_H_27_NO_5_	Dihydro-lycopsamine	+	−	+
13	10.65	[M+H]^+^	300.1804	156.1018, 139.0996, 138.0916, 122.0970, 120.0815	−0.5	C_15_H_25_NO_5_	Intermedine	+	−	+
14	12.82	[M-H]^−^	353.0882	191.0547, 179.0336, 135.0441	1.1	C_16_H_18_O_9_	Chlorogenic acid *	+	+	+
15	14.09	[M-H]^−^	385.0776	209.0301, 178.0294, 134.0360, 85.0305	−0.1	C_16_H_18_O_11_	Feruloylglucaric acid	−	+	+
16	14.73	[M-H]^−^/ [M+Cl]^−^/ [M+FA-H]^−^	343.1403/ 379.1169/ 389.1457	180.0784, 165.0551, 135.0442, 81.0352	1.3/ 1.0/ 1.0	C_16_H_24_O_8_	Cyclopentanehexanoic acid	−	+	+
17	15.28	[M-H]^−^	533.0924	209.0300, 191.0197, 179.0347, 85.0306	−2.4	C_24_H_26_O_14_	3,5-*O*-caffeoylquinic acid	−	+	+
18	15.45	[M-H]^−^	179.0362	135.0462	−6.8	C_9_H_8_O_4_	Caffeic acid	−	−	+
19	15.73	[M-H]^−^	533.0925	209.0301, 191.0189, 179.0342, 85.0306	−2.2	C_24_H_22_O_14_	1,5-*O*-caffeoylquinic acid	+	+	+
20	16.25	[M-H]^−^	533.0924	209.0306, 191.0201, 179.0332, 85.0312	−2.4	C_24_H_22_O_14_	3,5or1,5-*O*-caffeoylquinic acid isomer	+	+	+
21	16.54	[M-H]^−^	473.2021	265.1350, 247.1302, 203.1437, 119.0342, 93.0722	−1.6	C_22_H_34_O_11_	l-Ascorbic acid,6-[4-hydrogen (2*S*)-2-[(1-oxododecyl)oxy]butanedioate]	−	+	+
22	17.38	[M-H]^−^	367.1039	191.0560, 173.0445, 134.0373, 93.0354	1.2	C_17_H_20_O_9_	3-*O*-Feruloyl-quinic acid	−	+	+
23	17.89	[M-H]^−^/ [M+Cl]^−^/ [M+FA-H]^−^	415.1605/ 451.1369/ 461.1657	191.0548, 149.0436, 131.0350, 89.0252	−1.1/ −1.7/ −1.6	C_19_H_28_O_10_	Hexyl-3-(β-d-glucopyranosyloxy)-4,5-dihydroxybenzoate	+	+	+
24	18.05	[M+FA-H]^−^	463.2539	255.1961, 159.0329, 113.0246, 89.0265	−2.1	C_21_H_38_O_8_	3-*O*-[3-(Dodecyloxy)-2-hydroxypropyl]-L-ascorbic acid	−	−	+
25	18.46	[M+FA-H]^−^	377.1819	331.1754, 179.0551, 161.0443, 119.0345, 89.0251, 71.0154, 59.0162	0.5	C_16_H_28_O_7_	Menthol glucuronide	−	+	+
26	19.21	[M+FA-H]^−^/ [M+AcO-H]^−^	533.092/ 547.1078	209.0300, 191.0200, 179.0347, 85.0302	−3.2/ −2.8	C_23_H_20_O_12_	2-*O*-caffeoyl-3-*O*-feruloyltartaric acid	−	+	+
27	20.93	[M+H]^+^	465.1017	303.0490, 257.0436, 229.0493, 201.0544, 165.0181, 153.0182, 137.0236	−2.3	C_21_H_20_O_12_	Hyperoside *	−	+	+
28	21.35	[M+H]^+^	465.1019	303.0496, 257.0436, 229.0494, 201.0535, 165.0178, 153.0187, 85.0318	−1.8	C_21_H_20_O_12_	Isoquercitrin	−	+	+
29	21.49	[M-H]^−^	559.1078	397.0790, 235.0469, 179.0355, 161.0245, 135.0445	−2.7	C_26_H_24_O_14_	4,5-di-*O*-caffeoyl-2,7-anhydro-d-glycero-β-d-galacto-oct-2-ulopyranosonic acid	−	+	+
30	21.68	[M+H]^+^	595.1642	287.0552	−2.6	C_27_H_30_O_15_	Kaempferol 3-rutinoside	−	−	+
31	22.94	[M+H]^+^	595.1641	287.0548	−2.8	C_27_H_30_O_15_	Luteolin 7-*O*-rutinoside	−	+	+
32	23.63	[M+H]^+^	449.1073	287.0548, 258.0512, 213.0547, 165.0180, 153.0187, 121.0296, 85.0317	−1.2	C_21_H_20_O_11_	Trifolin	−	+	+
33	23.95	[M+H]^+^	551.1015	303.0496, 257.0440, 165.0185, 85.0319	−3.0	C_24_H_22_O_15_	Quercetin-*O*-malonyhexoside	−	+	+
34	25.01	[M+H]^+^	449.1070	287.0545, 241.0494, 213.0544, 153.0185, 121.0293, 85.0316	−1.9	C_21_H_20_O_11_	Kaempferol 7-*O*-glucoside	−	+	+
35	25.45	[M-H]^−^	515.1190	191.0554, 179.0346, 135.0447	−1.0	C_25_H_24_O_12_	Isochlorogenic acid A	−	+	+
36	25.92	[M+H]^+^	551.1011	303.0488, 257.0413, 165.0179, 85.0321	−3.7	C_24_H_22_O_15_	Quercetin 3-*O*-malonylglucoside	−	−	+
37	27.77	[M-H]^−^	515.1188	191.0564, 179.0353, 135.0453	−1.4	C_25_H_24_O_12_	Isochlorogenic acid B	−	+	+
38	29.13	[M+H]^+^	535.1057	287.0545, 258.0520, 213.0544, 153.0188, 85.0319	−4.7	C_24_H_22_O_14_	Luteolin 7-*O*-malonylglucoside	−	−	+
39	30.07	[M-H]^−^	455.1192	209.0272, 191.0223, 147.0279, 85.0305	−0.7	C_20_H_24_O_12_	β-d-Glucopyranosiduronicacid,3-hydroxy-4-(hydroxymethyl)phenyl, methylester,2,3,4-triacetate	−	+	+
40	32.61	[M+Cl]^−^/ [M+FA-H]^−^	435.2150/ 445.2438	179.0562, 143.0296, 89.0245	−1.2/ −1.1	C_21_H_36_O_7_	l-Ascorbic acid, 6-pentadecanoate	−	−	+
41	34.50	[M+NH_4_]^+^/ [M+Na]^+^	438.2112/ 443.1664	383.1466, 267.0990, 237.0881, 181.1025	−2.4/ −2.8	C_22_H_28_O_8_	3β-acetoxy-8β-(4′-hydroxy-tigloyloxy)-14-hydroxy-costunolide	−	+	+
42	34.67	[M-H]^−^/ [M+Cl]^−^/ [M+FA-H]^−^	447.2225/ 483.1991/ 493.2286	315.1919, 161.0455, 85.0308	−2.4/ −2.4/ −0.9	C_21_H_36_O_10_	Hexanedioicacid,1,1′-[(1-methyl-1,2-ethanediyl)bis[oxy(methyl-2,1-ethanediyl)]] ester	+	+	+
43	37.27	[M-H]^−^/ [M+Cl]^−^/ [M+FA-H]^−^	447.2228/ 483.1991/ 493.2283	315.1806, 191.0563, 161.0455	−1.7/ −2.4/ −1.5	C_21_H_36_O_10_	Hexanedioicacid,1,1′-[(1-methyl-1,2-ethanediyl)bis[oxy(methyl-2,1-ethanediyl)]] ester isomer	+	+	+
44	37.83	[M+H]^+^/ [M+NH_4_]^+^/ [M+Na]^+^	419.1689/ 436.1954/ 441.1497	267.2083, 243.0872, 197.0955, 181.0654	−2.7/ −2.7/ −5.2	C_22_H_26_O_8_	3β-acetoxy-8β-(4′-oxo-tigloyloxy)-14-hydroxy-heliangolide	−	+	+
45	37.88	[M-H]^−^/ [M+Cl]^−^/ [M+FA-H]^−^	355.2496/ 391.2257/ 401.2544	231.1793, 161.1863, 123.0797	1.7/ 0.1/ −0.2	C_20_H_36_O_5_	3-(hydroxymethyl)-1,12,14,15-tetrahydroxy-7,11,15,15-tetramethyl-2,6,10-hexadecatriene	−	+	+
46	38.14	[M+H]^+^	421.1844	227.1052, 209.0959, 199.1094, 181.1010, 165.0703	−3.1	C_22_H_26_O_8_	Eupalinolide C	−	+	+
47	38.42	[M+H]^+^	317.0657	302.0425, 168.0046, 140.0107, 137.0243	−1.2	C_16_H_12_O_7_	3-*O*-Methylquercetin	−	+	+
48	41.68	[M+H]^+^	421.1846	209.0919, 199.0769, 181.1015, 165.0704	−2.6	C_22_H_28_O_8_	Eupalinolide F	−	+	+
49	42.26	[M-H]^−^/ [M+Cl]^−^/ [M+FA-H]^−^	397.2594/ 433.2358/ 443.2646	59.0161	−0.4/ −1.0/ −1.0	C_22_H_38_O_6_	3-(hydroxymethyl)-1,13,15-trihydroxy-7,11,15-trimethyl-2,6,10-hexadecatrien-14-acetate	+	+	+
50	42.46	[M+H]^+^/ [M+NH_4_]^+^/ [M+Na]^+^	419.1693/ 436.1954/ 441.1511	267.2095, 243.0978, 225.0911, 197.0960, 165.0672, 154.0776	−1.8/ −2.7/ −2.0	C_22_H_26_O_8_	3β-acetoxy-8β-(4′-oxo-tigloyloxy)-14-hydroxy-costunolide	−	+	+
51	43.48	[M+H]^+^	331.0806	316.0570, 301.0367, 273.0399, 245.0441	−1.9	C_17_H_14_O_7_	Jaceosidin	−	+	+
52	44.38	[M+H]^+^/ [M+NH_4_]^+^/ [M+Na]^+^	419.1697/436.1955/ 441.1510	243.1078, 225.0942, 197.0979, 154.0779	−0.8/ −2.5/ −2.2	C_22_H_26_O_8_	Eupalinolide O	−	+	+
53	44.66	[M+H]^+^/ [M+NH_4_]^+^/ [M+Na]^+^	463.1944/480.2212/485.1769	227.1070, 209.0962, 199.0751, 181.1006, 165.0695	−4.0/ −3.3/ −2.7	C_24_H_30_O_9_	Eupalinolide A *	+	+	+
54	44.67	[M-H]^−^ [M+Cl]^−^/ [M+FA-H]^−^	397.2595/ 433.2361/ 443.2650	59.0158	−0.2/ −0.3/ −0.1	C_22_H_38_O_6_	3-(hydroxymethyl)-1,14,15-trihydroxy-7,11,15-trimethyl-2,6,10-hexadecatrien-13-acetate	−	+	+
55	47.10	[M+H]^+^	405.1913	183.1125, 153.0691	1.3	C_22_H_28_O_7_	hiyodorilactone B	−	+	+
56	47.42	[M+H]^+^	463.1949	227.1051, 209.0965, 181.1010, 165.0695	−2.9	C_24_H_30_O_9_	Eupalinolide B *	+	+	+
57	48.64	[M+H]^+^/ [M+NH_4_]^+^/ [M+Na]^+^	405.1901/ 422.2162/ 427.1713	195.0812, 183.1176, 168.0937, 153.0698	−1.7/ −2.7/ −3.3	C_22_H_28_O_7_	3β-acetoxy-8β-(4′-hydroxy-tigloyloxy)- costunolide	−	−	+
58	50.24	[M+H]^+^	405.1894	183.1219, 179.0852, 165.0693, 153.0705	−3.4	C_22_H_28_O_7_	Eupalinolide J	−	−	+
59	51.26	[M+NH_4_]^+^/ [M+Na]^+^	478.2069/ 483.1624	225.1020, 197.0946, 181.1020	−0.5/ −0.3	C_24_H_28_O_9_	Eupalinolide E	−	−	+
60	53.49	[M+H]^+^/ [M+NH_4_]^+^/ [M+Na]^+^	505.2057/ 522.2325/ 527.1879	209.0963, 199.0748, 181.1019	−2.2/ −1.7/ −1.6	C_26_H_32_O_10_	acetyl-eupalinolide A	−	+	+
61	55.84	[M+H]^+^/ [M+NH_4_]^+^/ [M+Na]^+^	505.2052/ 522.2320/ 527.1872	209.0956, 199.1117, 181.1009	−3.2/ −2.6/ −3.0	C_26_H_32_O_10_	acetyl-eupalinolide B	−	+	+

* Identified by reference standards; +: The compound is classified as a positive; −: The compound is classified as a negative.

**Table 2:** Linear relationships, limit of detection (LOD) and limit of quantitation (LOQ) of 5 analytes obtained using HPLC-PDA and UPLC-MS/MS.

Detection Methods	Analyte	Linearity			LOD (µg·mL^−1^)	LOQ (µg·mL^−1^)
		Calibration Curve	*r* ^2^	Range (µg·mL^−1^)		
HPLC-PDA	Hyperoside (**27**)	Y = 9870.2X − 11136	0.9999	3.47~222	1.16	3.47
HPLC-PDA	Eupalinolide A (**53**)	Y = 11271X + 37666	0.9999	8.30~531	2.77	8.30
HPLC-PDA	Eupalinolide B (**56**)	Y = 17940X − 15365	0.9999	7.79~510	2.67	7.97
HPLC-PDA	Chlorogenic acid (**14**)	Y = 5346.4X − 25388	0.9999	7.56~484	2.52	7.56
UPLC−MS/MS	Echinatine (**2**)	Y = 88139X + 255621	0.9973	0.000664~0.170	0.000050	0.000150

**Table 3:** Six batches of *Eupatorium lindleyanum* DC. samples.

Samples	Batch No.	Regions
S1	20220805	Jiangsu
S2	20220818	Jiangsu
S3	20220826	Jiangsu
S4	20220902	Jiangsu
S5	20220921	Jiangsu
S6	20221001	Jiangsu

“Batch No.” refers to the harvest time.

## Data Availability

The data used to support the findings of this study are available from the corresponding author upon request.

## Supplementary Materials

The following supporting information can be downloaded at: https://www.mdpi.com/article/10.3390/molecules29153551/s1, Figure S1. ESI-Q-TOF/MS(+) Spectra of Echinatine. Figure S2. ESI-Q-TOF/MS(+) Spectra of Hyperoside. Figure S3. ESI-Q-TOF/MS(+) Spectra of Eupalinolide A. Figure S4. ESI-Q-TOF/MS(-) Spectra of 3,5-O-caffeoylquinic acid. Figure S5. ESI-Q-TOF/MS(+) Spectra of 3-(hydroxymethyl)-1,14,15-trihydroxy-7,11,15-trimethyl-2,6,10-hexadecatrien-13-acetate. Figure S6. HPLC and MRM chromatograms of standard and sample solutions. Chlorogenic acid (**1**), Hyperoside (**2**), Eupalinolide A (**3**), Eupalinolide B (**4**), Echinatine (**5**).
