# Supplementary material for: Comprehensive Comparison of Three Different Medicinal Parts of Eupatorium lindleyanum DC. Using the RRLC-Q-TOF-MS-Based Metabolic Profile and In Vitro Anti-Inflammatory Activity
Source: Molecules. 2024 Jul 28;29(15):3551. doi: 10.3390/molecules29153551 (PMC11313985; doi:10.3390/molecules29153551)
Supplement: Supplementary file 1 [file molecules-29-03551-s001.zip › molecules-3115672-supplementary.pdf]

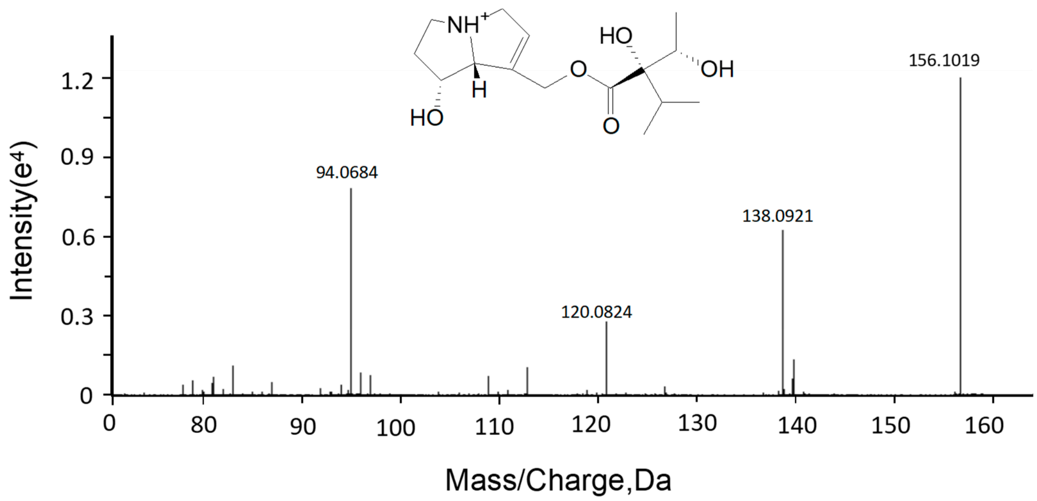

Figure S1. ESI-Q-TOF/MS(+) Spectra of Echinatine.

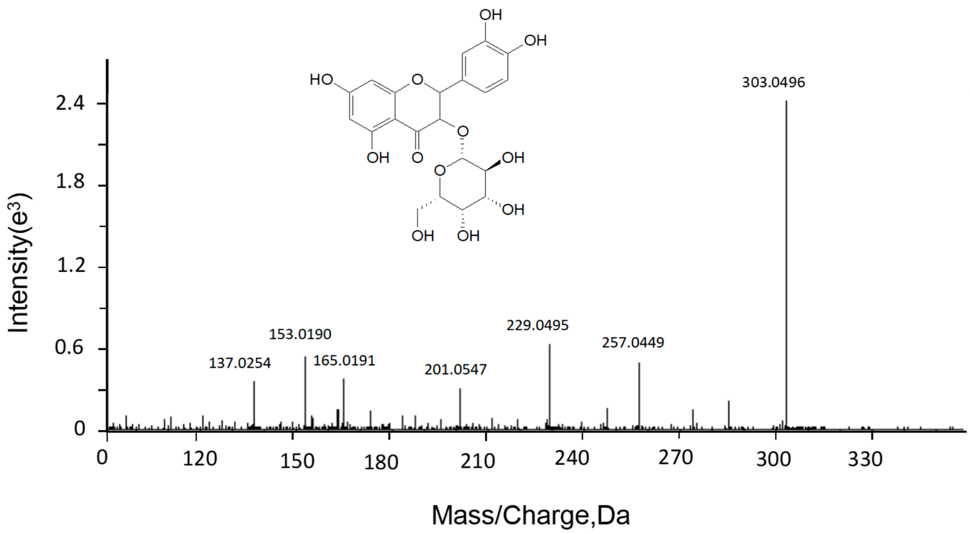

Figure S2. ESI-Q-TOF/MS(+) Spectra of Hyperoside.

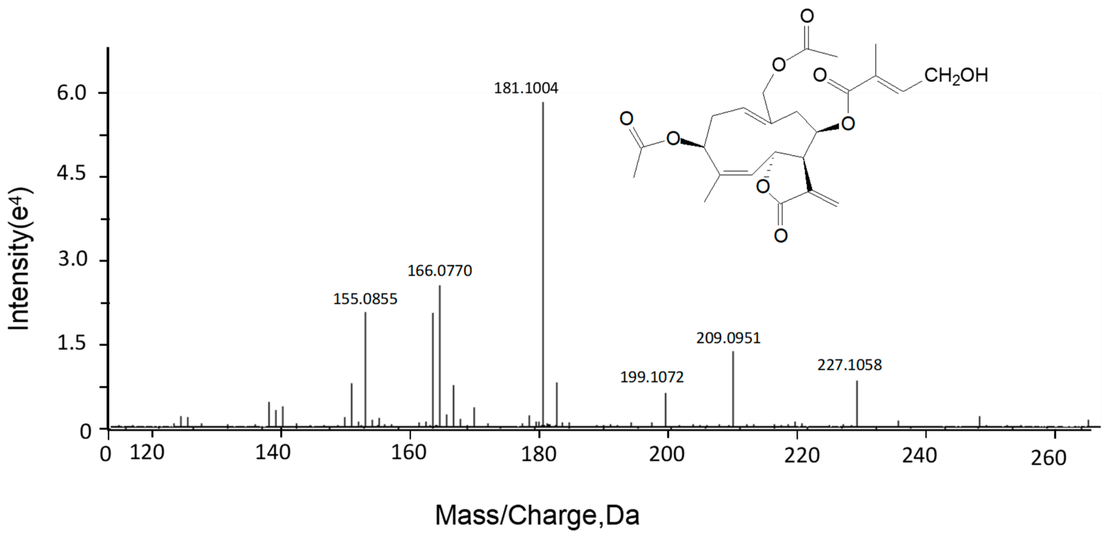

Figure S3. ESI-Q-TOF/MS(+) Spectra of Eupalinolide A.

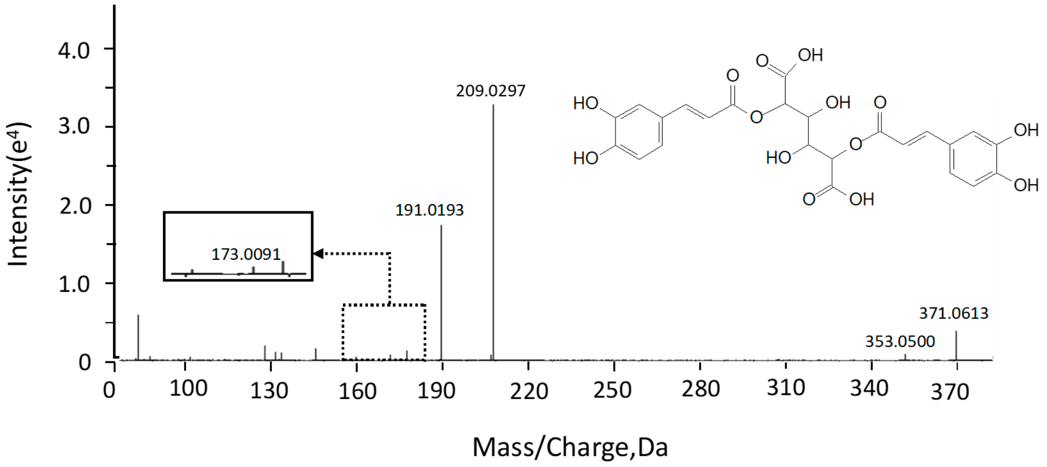

Figure S4. ESI-Q-TOF/MS(-) Spectra of 3,5-O-caffeoylquinic acid.

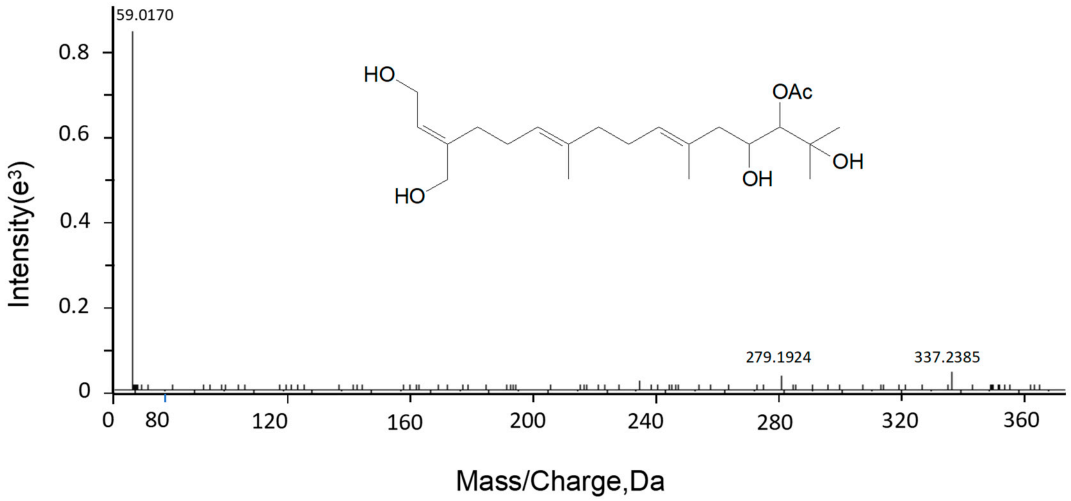

Figure S5. ESI-Q-TOF/MS(+) Spectra of 3-(hydroxymethyl)-1,14,15-trihydroxy -7,11,15-trimethyl- 2,6,10-hexadecatrien-13-acetate.

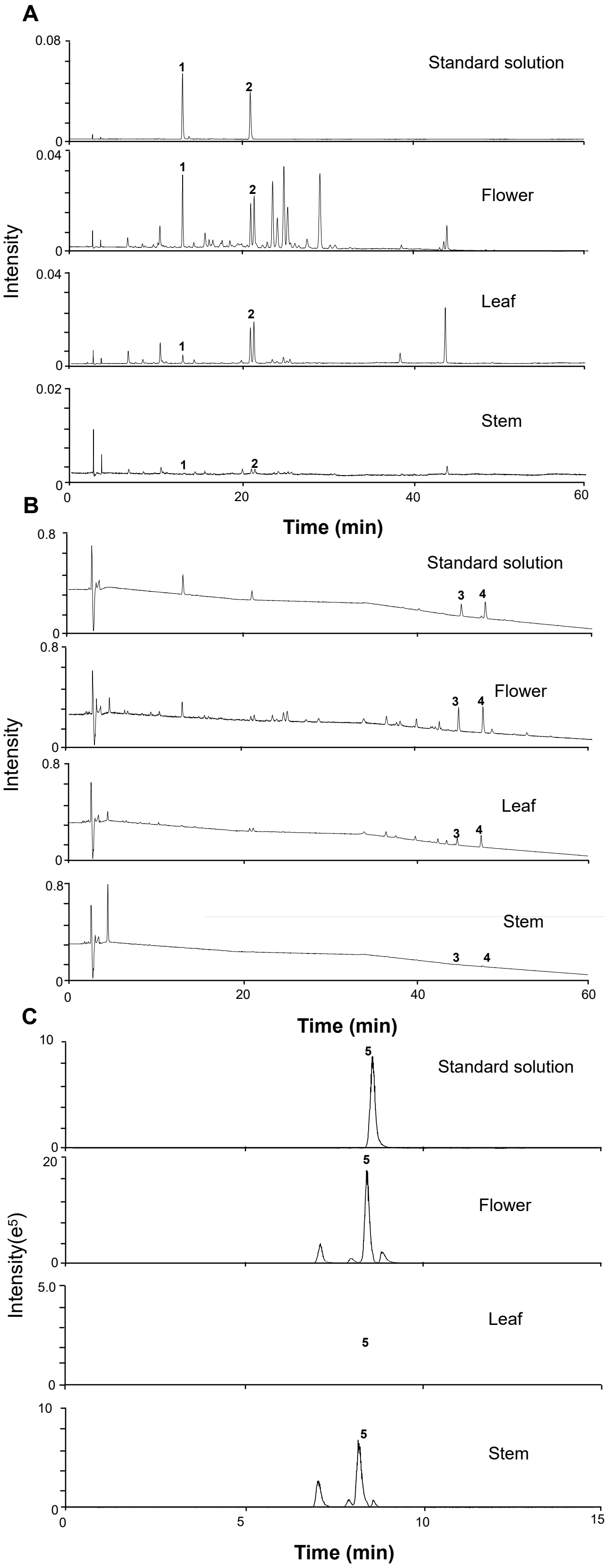

Figure S6. HPLC and MRM chromatograms of standard and sample solutions. Chlorogenic acid (**1**), Hyperoside (**2**), Eupalinolide A (**3**), Eupalinolide B (**4**), Echinatine (**5**).
